# Analgesic effect of auricular point acupressure for acute pain in patients with dementia: study protocol for a randomized controlled trial

**DOI:** 10.1186/s13063-022-06326-5

**Published:** 2022-05-14

**Authors:** Xiao-Min Chai, Hong-Yan Shi, Jun-Jun Zhang, Lei Wang, Hai-Xiang Gao, Ya-Liang Dai, Lu-Lu Gao, Jian-Qiang Yu, Yu-Xiang Li, Carol Chunfeng Wang

**Affiliations:** 1 School of Nursing, Ningxia Medical University, 1160 Sheng Li Street, Yinchuan, 750004 China; 2 Department of Geriatric Medicine and Special Medical, Ningxia Medical University General Hospital, 804 Sheng Li South Street, Yinchuan, 750004 China; 3 Department of Hematology and Oncology, International Cancer Center, Shenzhen Key Laboratory, Shenzhen University General Hospital, Shenzhen University Clinical Medical Academy, Shenzhen University Health Science Center, Xueyuan AVE 1098, Shenzhen, 518000 China; 4 The Third Middle school of Yinchuan, Yinchuan, 750001 China; 5 Department of Emergency, Yinchuan Second People’s Hospital, 684 Beijing Road, Yinchuan, 750000 China; 6 Department of Surgical, The First People’s Hospital of Yinchuan, 2 Li Qun West Street, Yinchuan, 750001 China; 7 School of Public Health, Xinxiang Medical University, 601 Jinsui Avenue, Xinxiang, 453003 China; 8 Department of Pharmacology, Pharmaceutical Institute of Ningxia Medical University, 1160 Sheng Li Street, Yinchuan, China; 9 School of Nursing and Midwifery, Edith Cowan University, 270 Joondalup Dr, Joondalup, WA 6027 Australia

**Keywords:** Acute pain, Analgesic, Auricular point acupressure, Dementia

## Abstract

**Background:**

Common and frequent as acute pain is, it is often underestimated and undertreated in older people with dementia in nursing homes and inadequate pain management remains an issue.

**Methods:**

The study is designed to be a randomized, sham-controlled trial and is underway in nursing homes located in China. A total of 206 dementia patients are being recruited from nursing homes in Yinchuan, China. They are randomly allocated to an intervention or a controlled group in a 1:1 ratio. The intervention group will be treated with true APP therapy, while the other group will receive APP at sham point stimulation therapy. The patients will be assessed at baseline (T0), at 5 min during performing the intervention (T1), and at 5 min after completion of the intervention (T2). The primary outcome is the level of pain relief at T1 and T2. Physiological parameters, side effects and additional use of analgesics during the procedure, satisfaction from caregivers, and acceptance of patients are evaluated as secondary outcomes.

**Discussion:**

The results of this study are expected to verify the analgesic effect of APP for acute pain in patients with mild dementia in nursing homes. It has the potential to prompt APP therapy to be implemented widely in dementia patients with acute pain in nursing homes.

**Trial registration:**

Chinese Clinical Trial Registry ChiCTR2100047932. Registered on 27 June 2021. Currently, patient recruitment is ongoing. Recruitment is expected to take place from December 2020 to December 2021.

## Introduction

For residents in the nursing home, dementia and pain have aroused great public concern and a lot of discussions in the global medical policy, research and education fields for many years [1]. Pain is a common experience for many older adults with dementia in nursing homes. It is estimated that the prevalence of pain in dementia patients residing in the nursing home is 19–83%, and the pain they are suffering is difficult to assess and inadequately treated [2–4]. There is also a guideline that indicates that the incidence of acute pain is as high as 75% in elderly care facilities, but even higher for chronic pain [5]. Furthermore, uncontrolled pain causes many detrimental consequences for patients with dementia in nursing homes, such as distressing psychological, social isolation, daily activity and function limitation, sleep disturbances, hospital stays prolonging, and medical expenses increasing [6–8]. In particular, acute pain in elderly patients can have a serious impact on psychology and even lead to an attempt to suicide [8].

Acute pain is common and frequent, but it is often underestimated and undertreated in elderly people with dementia in nursing homes. Therefore, poor pain management in this population remains a problem [4]. Complications of acute pain have been well documented. However, few clear guidelines are currently available on the optimal therapies to measure and treat acute pain in patients with dementia in nursing homes [6]. Pharmacological and non-pharmacological modalities have been practiced to manage acute pain and its associated complications in older adults with dementia over the past decade. It is advised that pharmacological treatment to control acute pain in the elderly should generally follow a three-step scheme: paracetamol, non-steroidal anti-inflammatory drugs (NSAIDs), opioids, and the other adjuvant drugs. Although paracetamol is recommended worldwide as a first-line pain reliever for patients with dementia in nursing homes, there are limited reports on its analgesic effects [9]. Several studies have also evidenced that elderly persons with dementia are prescribed fewer NSAIDs and other analgesics than those without dementia in nursing homes, and there are not many opportunities to consider the use of strong analgesics [9, 10]. Patients with dementia are prescribed opioid analgesics, even though the doses they were allowed to take are one-third of those who are cognitively intact elder adults, especially in the nursing home residents with dementia [11, 12]. There is little evidence of the effectiveness of adjuvant drugs, such as anti-inflammatory drugs, anticonvulsants, and antidepressants, for relieving acute pain in older adults with dementia in nursing homes [11]. Furthermore, they are more prone to have drug-related side effects [7]. These factors also contribute to poor acute pain control in patients with dementia in the nursing home.

Therefore, it is important to seek non-drug therapies for controlling dementia patients with acute pain. Traditional Chinese medicine (TCM) theory holds that non-pharmacological methods, such as acupoint acupressure, acupuncture, massage, psychotherapy, and electroshock therapy, play a positive and important role in the treatment of acute pain. Non-pharmacological interventions have become the priority for patients with acute pain due to fewer side effects, non-invasive, and better safety [13]. According to TCM theory, the ear is where the body’s meridians (the main twelve meridians: six of which are yin meridians and six yang meridians) connect and where the essential Qi converges. So, stimulating auricular points can activate Qi and blood circulation, dredge the meridians, and then achieve the purpose of analgesia [14]. Stimulating auricular acupoints can regulate the secretion of central neurotransmitters, thereby activating the analgesic system in vivo. In other words, it exerts a pain-relieving effect by activating the downward pain inhibitory pathway and thus inhibiting the upward pain pathway in the brainstem [14]. Systematic review and meta-analysis also reported that APP showed promising results in pain relief in the experimental group compared with the control group [15, 16]. APP therapy has been widely applied in various types of pain, such as acute postoperative pain, pain related to dental, and musculoskeletal pain in older adults, and as an adjunct analgesic among cancer patients [15–17]. A dearth of the study explored that APP therapy is applied to control acute pain in nursing home residents with dementia. Therefore, a randomized, sham-controlled trial was designed to evaluate the analgesic effect of APP for senile dementia patients with acute pain in nursing homes.

## Methods

### Study design

**Fig. 1:**
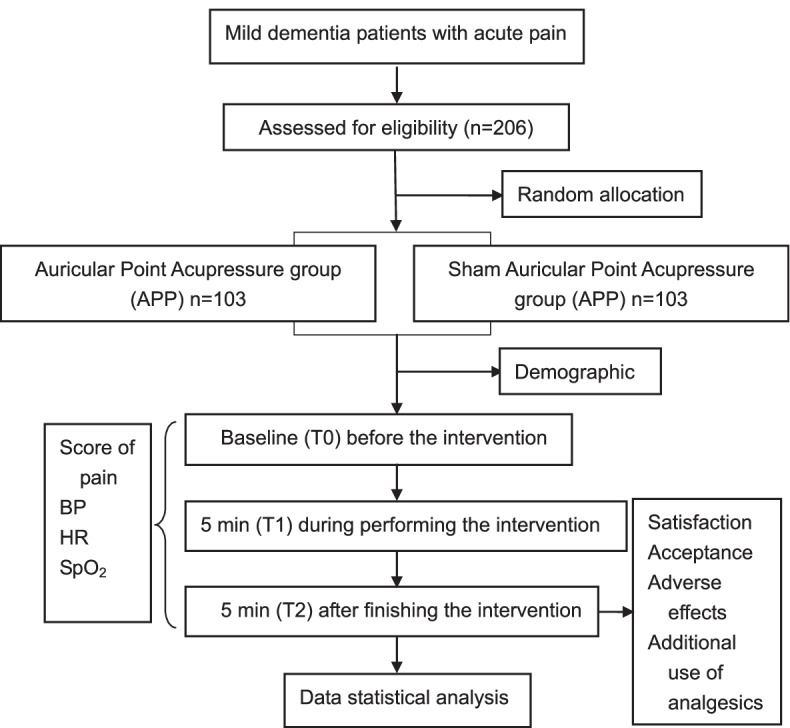
Study design framework. BP, blood pressure; HR, heart rate; SpO_2_, oxygen saturation; APP, auricular point acupressure; Score of pain, the Face Pain Scale Revised

This study is a multi-center and single-blind, randomized, sham-controlled clinical trial. It aims to compare and analyze the one-time analgesic effect between the intervention group and the sham-controlled group (Fig. 1, Table 1) for acute pain in patients with dementia. This trial, superiority framework, was designed on the basis of SPIRIT guidelines and the Consolidated Standards of Reporting Trials (CONSORT) statement [18, 19]. The Ningxia Medical University Ethics Committee has given its official approval for the study protocol, which has been registered at the Chinese Clinical Trial Registry (No.: ChiCTR2100047932). Each patient will be given a detailed explanation by the researcher including the purpose, benefits, and potential risks of the study. Moreover, they can withdraw from the study at any time, and this will not affect medical services other than the research. Subsequently, the qualified recruits who signed informed consent entering the study will receive APP or shamed APP treatment by an investigator, a master candidate in nursing science with APP professional training. It is needed to make important protocol modifications to reduce underpower of the study protocol and increase patient safety. We will communicate with the Data Management and Safety Monitoring Committee (DMSMC), all participants, and the Ethics Committee.

**Table 1:** Schedule of enrollment, interventions, and assessments

Procedure	Study period			
	Enrolment	Post-allocation		
	Dementia patients with acute pain	T0	T1	T2
Enrolment				
Eligibility screen	×			
Informed consent	×			
Allocation	×			
Interventions				
Control group			×	×
Treatment group			×	×
Assessments				
Face pain scale revised	×	×	×	×
Blood pressure		×	×	×
Oxygen saturation		×	×	×
Heart rate		×	×	×
Satisfaction				×
Acceptance				×
Side effect			×	×
Additional use of analgesics during the intervention				×

### Study setting

This randomized controlled trial is being conducted at three nursing homes in Yinchuan, China. These institutions are urban and public in the region, with approximately 2000 permanent residents.

### Patients

All 206 patients who met the inclusion and exclusion criteria are being recruited from the three nursing homes. Recruitment is expected to take place from December 2020 to December 2021. The recruits with informed consent before entering research will be randomly allocated to an intervention or a sham-controlled group in a 1:1 ratio. Furthermore, it is feasible to have enough patients to meet the target sample size in three nursing homes. The inclusion criteria include the following: (1) aged 60 or above with speaking Chinese, (2) dementia was diagnosed by the Diagnostic and Statistical Manual of Mental Disorders (DMS-V) criteria [20], (3) diagnosis of mild dementia based on the Montreal Cognitive Assessment (MoCA) [21], (4) acute pain and a score of pain ≥ 4 based on the Face Pain Scale Revised (FPS-R), (5) able to be monitored by pulse oximetry, (6) understanding the meaning of the FPS-R, and (7) volunteering to participate in the study and signing the informed consent (caregivers, patients, and/or family members). The exclusion criteria are as follows: (1) moderate or severe dementia, (2) medical contraindication for ear acupressure (inflammation, ulcers, frostbite in the ear), (3) history of allergy to adhesive tape and alcohol, (4) critically ill patients who have no response to the effectiveness and safety of new treatments (serious heart, brain, liver, kidney, or hematopoietic system diseases), and (5) patients who received drugs for pain management and other interventions. Once the subjects were enrolled, written informed consent from all patients and their caregivers was obtained by the researchers.

### Interventions

The intervention group will be planned to receive auricular-plaster therapy, with one piece of Semen Vaccariae® (about 2 mm in diameter, Taicheng Technology and Development Co., Ltd., Shanghai, China) attaching to the center of a piece of medical adhesive tape (6 mm × 6 mm). This study initially selected four active acupoints including shenmen (TF4), subcortex (AT4), adrenal (TG2P), and two auricular points corresponding to pain sites based on the Nogier auricular point diagram for managing pain [22]. A researcher will bring the required items to the participants’ bedside and assist them in taking the position that is easy to operate. Subsequently, the researcher holds the helix with the left hand and identifies sensitive points by virtue of an ear probe with the right. The medical adhesive tapes with Semen Vaccariae® will be pasted on the selected auricular points with forceps by the researcher after checking and sterilizing the skin of the patients’ ears using 75% alcohol. Then, each auricular point will be pressed gently 2 times lasting 1 min each time. There is no time interval during the operation unless adverse reactions occur or until finishing the procedure. The pressure should be moderate and enough within individual’s endurance (the subject feels swelling pain, numb, and warm sensation). After the whole intervention, these tapes will be removed. However, these five points (kidney, CO10; ocuiput, AT3; internal ear, L06; external ear, TG1u; adrenal gland, TG2P) were selected in the control group. The main effect of these five points was to delay hearing loss; its procedures are described in the intervention group. However, the subject simply experiences the warm sensation in their ears. Relevant concomitant care is permitted during the trial. Although adverse reactions associated with auricular acupressure are rare, in this study, if they occurred (side effects occur during the implementation, they are short-lived and mild [15, 16]) or the participant’s requirements that the intervention they receive be discontinued, researchers can determine whether the trial will continue. Furthermore, the reasons for the withdrawal will be logged. Strategies to improve adherence to intervention protocols will be applied in *Trials*. Prior to the protocol, researchers will describe the method and duration of the intervention and the potential risk involved to the participants in detail [23]. In the intervention, there are many patients’ physiological parameters monitored by the data collector. A supervised compliance form (SAF) for this study protocol is necessary to collate patient compliance [23].

### Randomization and blinding

Sequence numbers of the patients with dementia will be generated by a computer-produced random list, which is performed by a statistical expert from Ningxia Medical University. But she is independent of the trial. The randomization schedule in a sealed envelope will be kept in a dedicated research office. There are many strategies to ensure the concealment of data distribution and minimize selection bias as well as create a double-blind environment. It will be blind to the randomized lists for participants (e.g., other researchers, patients, and caregivers) except the investigator responsible for conducting the intervention [24]. The project manager can suspend blinding until all the trials have been carried out according to the rules. The research team is responsible for reducing the rate of loss of intervention. Prior to the trial, the researcher went into the nursing home and lived and worked with the caregivers of the elderly to learn about the patient’s lifestyle. Meanwhile, they obtained their and their caregivers’ support and cooperation. Patients and their caregivers will be presented face to face with detailed information, which includes intervention process, collection and use of data and biological specimens, benefits, and potential adverse reactions from the trial [23]. But patients also have the right to withdraw from the study at any time without affecting subsequent care. In case patients and (or) their caregivers decide to quit the trials, data and biological specimens will be destroyed. It has been also documented in the informed consent form.

### Measurement

Each patient’s demographic data (age, sex, nationality, educational background, marital status, occupation before retirement, personal income per month, history of taking medicine, chronic case history) will be recorded by a self-designed questionnaire. Other information including physiological parameters (heart rate, blood pressure, and oxygen saturation), sore spot, pain score, adverse effects, the use of analgesics in the intervention, satisfaction of caregivers, and acceptance of patients, will be more rigorously documented. The MoCA is made to identify the severity of dementia among the patient. And locality of the pain will be shown clearly according to the Brief Pain Inventory Chinese version (BPI-C) [25]. The FPS-R with great reliability and validity will be elected to assess pain intensity, and three physiological parameters are monitored with electronic sphygmomanometer (OMRON, HEM-7120) and Fingertip Oximeter (PC-60B). The data required for the study include pain score, noninvasive monitoring of blood pressure, heart rate collected at baseline (before performing the intervention, T0), at 5 min during performing the intervention (T1), and at 5 min after the intervention finished (T2) as well as digital monitoring of oxygen saturation. The intervention process of APP should be closely monitored, even if there are no reports of its side effects. And it will be recorded in detail and properly handled for any adverse reactions observed. The use of analgesics in the intervention also will be carefully recorded to enhance analysis of the safety and analgesic effect of APP. Satisfaction from caregivers will be surveyed via a 5-point satisfaction scale (5, very satisfied; 4, satisfied; 3, uncertain; 2, dissatisfied; 1, very dissatisfied) at T2 [26]. However, acceptance of patients will be measured by just asking them if they would like to accept the APP treatment once again.

### Outcome measurement

#### Primary outcome measure

The level of pain relief at T1 and T2 will be considered as the primary outcome. It is accurately recorded by a researcher in charge of recording on the basis of the FPS-R.

#### Secondary outcome measures

Secondary *outcome measures* will consist of physiological parameters, any adverse reactions observed, satisfaction from caregivers, acceptance of patients, and additional use of analgesics during the intervention. The duration of the intervention will also be included in the secondary outcomes.

### Sample size estimation

After obtaining the ethical approval of our study protocol, a preliminary experiment with a sample size of 30 was conducted in Nursing Home, Yinchuan, in January 2020. The results of the pilot study indicated that the FPS-R score of the control group and the intervention group was 5.97 ± 1.81 and 4.79 ± 1.96, respectively. We have consulted a statistician from the School of Medical Statistics and Epidemiology at Ningxia Medical University, who finished the randomization list of the recruits. With a type 1 error rate of 0.05 (*α* = 0.05, two-tail) and a 90% power (*β* = 0.10), the sample size of the study scheme was calculated by the statistical expert, which was 86 patients in each group. According to our study design and considering the particularity of this research subject (they are less likely to complete the entire experiment), we assume a 20% drop-out rate. The total sample size of 206 cases (per group was 103 cases) is required to reduce the underpower of the study.

### Management and safety monitoring

Prior to the study, research team members will receive a training program, which will cover the research design, procedure, evaluation of outcome measures, randomization, data collection, and data monitoring. For example, data recorders will be trained on how to collect and manage data to ensure the objectivity, quality, and safety of data as far as possible. Patients were anonymous throughout the trial. All subject’s personal information will also be stored in a separate envelope. It will then be saved in a Microsoft Access (Microsoft Office 2010, Redmond, WA, USA) database, which is password protected [23]. A separate code was generated by pre-randomization and kept by the project manager. It is a unique identifier that identifies anonymized data. With the consent of the project leader, only members of the research team have access to the collected information for a reasonable reason [24]. Another Microsoft Access database will be used to store all trial data. It can only be got by the data collectors, the investigator, and the DMSMC. It is necessary although the APP treatment was the least risky. It consisted of the project manager, principal researcher and physician of each sub-center, and experts from the Ethics Committee of Ningxia Medical University. They are primarily responsible for the quality control of clinical trials, including but not limited to the data collection and management and uniform training for investigators. Regular monitoring and auditing data will be also required. It should be also noted that the trained data collectors and investigators are fixed and that data quality control is carried out through double-entry bookkeeping by them [23, 24]. In addition, information on patients with withdrawal will not be included in the final analysis, and other researchers will investigate what led them to withdraw from the trial. According to previous studies, the side effects of this intervention have been reported rarely. Conducting an interim analysis or stopping rules will not be applied to the current study. All of the study subjects were elderly patients, so they will be closely monitored as part of the standard of care before, during, and after study participation.

### Data statistical analysis

On the basis of the intention-to-treat (ITT) principle, SPSS version 22.0 (Chicago, IL, USA) will be recommended to run the data statistical analysis on both the real APP group and the sham APP group. Information on all patients in the random list should be included in the final analysis. If data is missing, the missing data imputation should be used to analyze the validity of the statistical analysis. The statistical description of categorical variables (the population demographic data) will be mainly presented by the relative number (rate, proportion, and ratio). The continuous variables (repeated measurement data of three physiological parameters) will be compared by analysis of variance (ANOVA) or the Kruskal-Wallis test (or chi-squared tests) for the two groups. Other categorical variables (data on satisfaction from caregiver, acceptance from patient, and additional use of analgesics) will be described by the nonparametric test (Wilcoxon’s rank sum test). A two-sided *P* value < 0.05 will be considered to be statistical significance. Therefore, subjects’ personal information, such as nationality, education, and medication history, will be used to consider whether to perform statistical analysis in subgroups.

## Discussion

Every patient is endowed with his or her basic and undeniable right of eliminating pain. Meanwhile, within the scope of our expertise, medical professionals have an obligation to consider giving patients reasonable pain management [27], but many studies have shown that pain in people with dementia is often underestimated or even ignored in nursing home settings [6, 7, 28]. This results in inadequate treatment of pain. In addition, the toxic side effects caused by analgesic drugs, such as acute respiratory depression, addiction, ulcers, and hemorrhage, make Chinese medical staff and patients concerned about their use [28]. This study aims to find a better evidence-based and alternative therapy for dementia patients with pain in the nursing home, in China. The study by Xia et al. showed a significant reduction in pain scores in patients with axial neck pain after anterior cervical discectomy and fusion in the APP group compared with the sham APP group [29]. However, there is also a study that demonstrated that no significant difference in pain score reduction was found in acute postpartum perineal pain between the intervention and control groups [30]. In this study, we will illuminate the role of APP treatment in managing acute pain in patients with dementia in nursing homes.

What we know is that our study protocol is the first randomized controlled trial to identify the analgesic effectiveness of APP for acute pain in patients with dementia in nursing homes, China. It has the potential to develop preliminary evidence-based guidance for relieving pain in patients with dementia in nursing homes if the analgesic efficacy of APP appears beneficial and safe. We hope that the results generated by the trial will be published in peer-reviewed journals.

One of the biggest limitations of this study is that it only evaluates the one-time efficacy of the intervention. But this trial really only evaluates the analgesic effect of APP therapy for dementia patients with acute pain in nursing homes. We also plan to move forward with the study after certifying the analgesic effect of APP therapy.

## Data Availability

Trial data are available on reasonable request to the principal investigator of the study.

## Abbreviations

APP: Auricular point acupressure
ANCOVA: Analysis of covariant
BPI-C: Brief Pain Inventory Chinese Version
CONSORT: Consolidated Standards of Reporting Trials
DMSMC: Data Management and Safety Monitoring Committee
DMS-VI: Diagnostic and Statistical Manual of Mental Disorders-VI
FPS-R: Face Pain Scale Revised
ITT: Intention-to-treat
MoCA: Montreal Cognitive Assessment
NSAIDs: Non-steroidal anti-inflammatory drugs
SAF: Supervised compliance form
TCM: Traditional Chinese medicine
